# Transverse tendon stiffness is reduced in people with Achilles tendinopathy: A cross-sectional study

**DOI:** 10.1371/journal.pone.0211863

**Published:** 2019-02-20

**Authors:** Evan Finnamore, Charlotte Waugh, Lyndal Solomons, Michael Ryan, Christopher West, Alexander Scott

**Affiliations:** 1 Centre for Hip Health and Mobility, Vancouver Coastal Health Research Institute, Vancouver, Canada; 2 Department of Physical Therapy, Faculty of Medicine, University of British Columbia, Vancouver, Canada; 3 Department of Biomedical Physiology and Kinesiology, Simon Fraser University, Burnaby, Canada; 4 Southern Medical Program, Department of Cellular and Physiological Sciences, University of British Columbia (Okanagan), Kelowna, Canada; University of Canberra, AUSTRALIA

## Abstract

**Objectives:**

The objective of the current cross-sectional study was to examine Achilles tendon transverse stiffness in a group of recreational runners with Achilles tendinopathy, in comparison to an asymptomatic group of runners with similar training history. We also aimed to determine the between-week intra-individual reliability of this measurement technique.

**Design:**

Cross-sectional cohort study.

**Methods:**

A hand-held dynamometer was used to assess the transverse stiffness of the Achilles tendon (AT) in twenty-five recreational runners. In ten people with midportion Achilles tendinopathy (5 men, 5 women), measurements were taken directly over the most symptomatic location. In 15 people who were free of AT symptoms (7 men, 8 women), measurements were taken at an equivalent location on the tendon. Participants returned after one week to determine measurement reliability (intra-class correlation coefficient/ICC and minimum detectable change/MDC95). We also collected information about people’s tendon loading activities, tendon thickness (ultrasound mesaurement), and symptoms (Victorian Institute of Sports Assessment–Achilles / VISA-A score).

**Results:**

The AT transverse stiffness was lower in people with Achilles tendinopathy (777 N/m ± 86) compared to those who were asymptomatic (873 N/m ± 72) (p < 0.05). AT transverse stiffness was negatively correlated with age and tendon thickness, and positively correlated with VISA-A score and waist circumference. Reliability was good, with ICC of 0.81 in people with tendinopathy and 0.80 in healthy controls, and an MDC95 of 118 and 87N/m in these two respective groups.

**Conclusions:**

Transverse Achilles tendon stiffness can be reliably measured in people with midportion Achilles tendinopathy, and appears to be lower in people who are older, more symptomatic, and with more extensive tendon thickening. The potential clinical utility of monitoring tendon stiffness in the management of tendon injuries merits further study.

## Introduction

We recently assessed the feasibility of measuring the transverse stiffness (*k*_*TR*_) of the Achilles and patellar tendons.[1] We found that a commercially available unit (MyotonPRO, Myoton AS, Estonia) accurately measures this parameter, and in adult tendon tissue the presence of skin beneath the probe tip has a negligible influence on stiffness. In a field study of 66 recreational runners, men displayed stiffer tendons than women, and more highly trained individuals had increased Achilles stiffness compared to those who were less highly trained. These results encouraged us to explore the potential utility of measuring Achilles tendon transverse stiffness in people with symptomatic tendinopathy.

Tendon stiffness is traditionally measured in the longitudinal direction (e.g. tensile stiffness, *k*_TE_), in accordance with its line of pull [2]. Stiffness is crucial to the function of the Achilles tendon [3], and it is known to be reduced, on average, in people with Achilles tendinopathy [4]. Tendon tensile stiffness is a difficult parameter to measure routinely in the clinic, requiring synchronized data collection from real-time ultrasound and dynamometry. As an alternative, the assessment of tendon transverse stiffness has been considered, and a rapidly growing body of work is emerging in this area employing elastography[5] and shear wave tensiometers[6]. Using shear-wave elastography, Coombes et al found that transverse Achilles tendon stiffness was reduced in the tendinopathic Achilles compared to healthy tendon, indicating that the symptomatic Achilles tissue was softer (more compliant) in the painful region [7]. This observation, although preliminary, is consistent with the pathophysiology of injured Achilles tendon, which is typically characterized by increased type III collagen and elevated blood flow (changes which would be expected to lead to a softer tissue with reduced ability to resist load deformation)[8]. A deficit in tissue load tolerance could contribute to a scenario of failed healing and repeated provocation of nociception (mechanical hyperalgesia).

The objective of the current cross-sectional study was to compare Achilles tendon transverse stiffness (*k*_*TR*_) in a group of recreational runners with Achilles tendinopathy, in comparison to an asymptomatic group of runners with equivalent training history. We also aimed to determine the between-week intra-individual reliability of *k*_*TR*_ in the same participants.We hypothesized that stiffness would be lower in the tendinopathy group,[4] and would be negatively correlated with age and waist circumference [9] [10] and positively correlated withVISA-A (Victoria Institute of Sports Assessment—Achilles)[11].

## Methods

This study was approved by the Clinical Research Ethics Board, University of British Columbia. Certificate # H16-03381.

### Study design

The study was designed and reported using the Strobe checklist for reporting of cohort studies [12]. This was a cross-sectional study comparing the Achilles tendon transverse stiffness in recreational runners with, or without, current Achilles tendinopathy. The reliability (intraclass correlation coefficient/ICC and minimum detectable change/MDC95) of *k*_*TR*_ were evaluated by testing on two occasions separated by one week by the same evaluator. The average tendon measurement was taken from each individual (i.e. side-to-side differences were not examined, due to potentially complex interactions between leg dominance and asymptomatic bilateral tendon changes)[13,14].

### Participants

A purposive sample of recreational runners with or without Achilles tendinopathy was recruited by advertising through local running groups. The inclusion criteria for both groups were age (18–50), fluency in English, and running frequency (≥ 1 run per week for the past year)[15]. Achilles tendinopathy participants must have been diagnosed by a healthcare professional with chronic midportion Achilles tendinopathy, have been symptomatic ≥ 3 months [16], and demonstrate tenderness on palpation, pain with tendon loading, and typical ultrasound findings [17]. Controls had to be symptom-free, with no history of Achilles pain or injury. Exclusion criteria for both groups were pregnancy, major surgery in the past 3 months, previous corticosteroid injections or recent fluoroquinolone use, insertional Achilles pain, and diabetes or other medical or orthopedic conditions which could affect tendon properties [18].

We attempted to limit sampling bias by recruiting the comparison group from the same running communities as the tendinopathy group. To limit sex/gender and age bias, we decided *a priori* to assemble equivalent sized tendinopathy and control groups with equal proportions of males and females and of similar age. To limit potential bias introduced by different levels of physical activity, we recruited individuals for whom running was the main activity and attempted to quantify this by self-report, but acknowledge that there is not currently a valid way of evaluating a person’s volume of Achilles tendon loading.

### Study visits

Each subject attended two data collection appointments separated by one week, at the Centre for Hip Health and Mobility at Vancouver General Hospital, Vancouver BC, Canada. Body mass and height (Seca 284, Seca, Hamburg, Germany) and waist circumference (just superior to the iliac crest) were measured on the first of two appointments. Other relevant information such as age, sex assigned at birth, weekly running volume and frequency, leg dominance (limb used to kick a ball), years of running experience and VISA-A [11] were self-reported. One investigator collected ultrasound scans (see below) and inquired about the frequency and duration of running on the first appointment. On the first and second appointments, *k*_*TR*_ was measured (see below). The assessment of all variables, including VISA-A, was the same for both groups.

### Achilles tendon stiffness measurement

**Fig 1:**
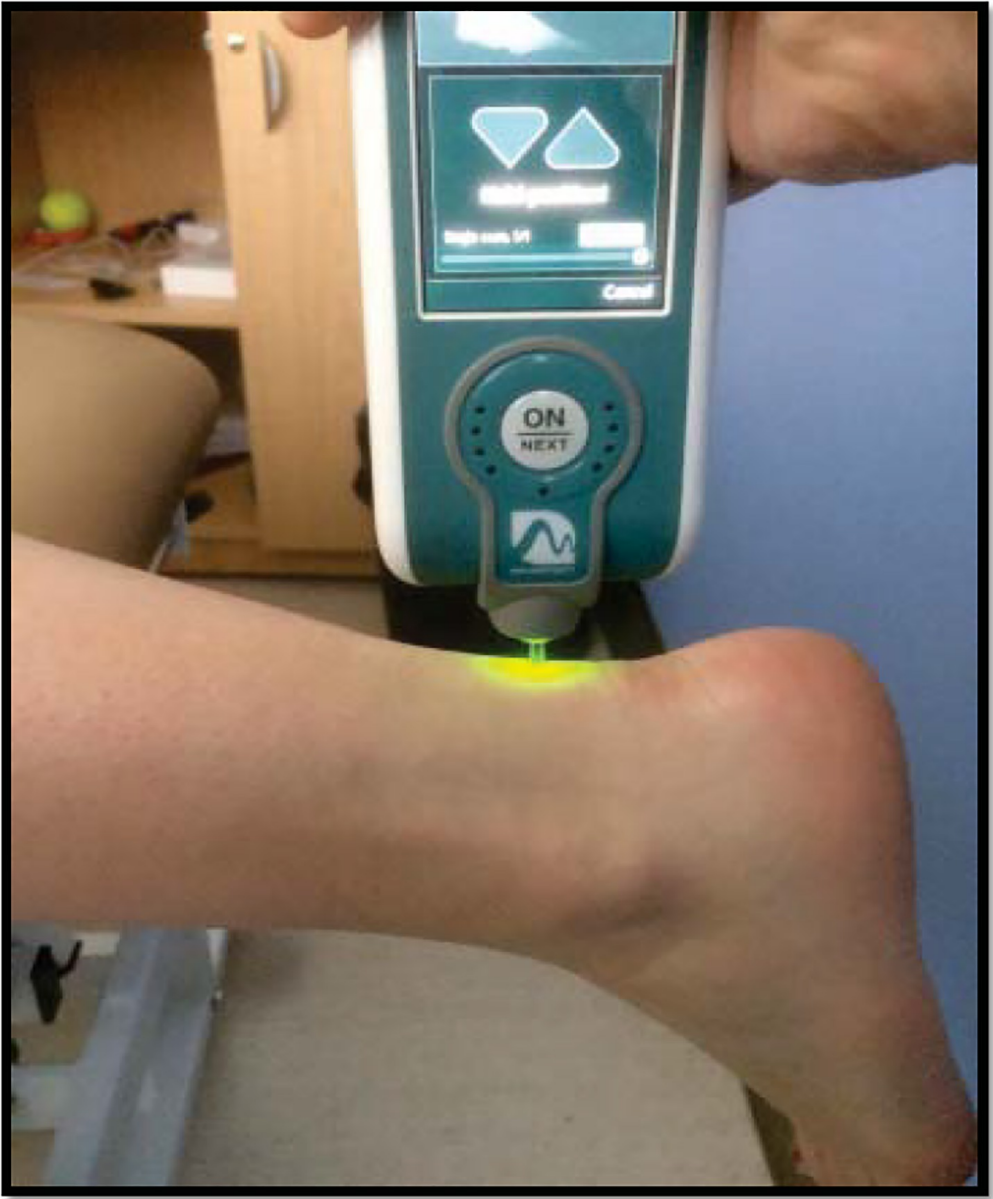
Testing position for transverse Achilles tendon stiffness measurement.

Subjects lay prone on a treatment plinth with the calf muscles relaxed and the foot hanging freely, as previously described [1]. We did not attempt to influence the resting length of the tendon by requiring the ankle to be in a particular position, but simply instructed the person to relax their leg. In Achilles tendinopathy participants, the MyotonPRO probe tip was placed perpendicular to the Achilles tendon (Fig 1) directly over the site of maximal tenderness, which corresponded in each case to the location of maximal thickening. A few patients reported current or previous bilateral symptoms, but only the worst side was measured; all patients could clearly identify a worst symptomatic side without hesitation, and this was on the non-dominant Achilles tendon in 9 / 10 tendinopathy participants. The distance of the testing location from the calcaneus was recorded, and the average (3.7cm) was used as the test site for control participants. Stiffness has been shown to vary between dominant and non-dominant legs [14], therefore in controls the Achilles tendon on the non-dominant leg was used for comparison. The probe tip was pre-loaded to 0.18N [19], and then five automated, consecutive stiffness measurements were taken in quick succession (15ms tap time, 0.4N impulse, separated by 0.7s); the average of these five measures was displayed by the Myoton unit. If the coefficient of variation of the five measurements was above 3%, a red light warning appeared on the Myoton unit, and the values were discarded and five measurements were retaken. If the coefficient of variation of the five measurements was 3% or less, the average stiffness value displayed by the Myoton was recorded. The situation of high coefficient of variation occurred rarely, and was usually associated with movement artefact (patient or tester). The entire procedure was repeated on visit 2.

### Ultrasound scans

Ultrasound scans were conducted as previously reported [20]. Six hundred consecutive transverse images of the Achilles tendon were captured every 0.2 mm over a length of 12 cm using a 10-MHz linear-array transducer (Smartprobe 10L5; Terason 2000, Teratech, USA) attached to an(UTC Technologies, Kruisstraat, Netherlands) automated tracker. The scanner was held stationary in a clamp, and the foot was braced against the wall at one of three fixed angles, as required to orient the tendon parallel to the ultrasound probe. If 0° of dorsiflexion is defined as a 90° angle between foot and shank, then the angles were 0°, 10° or 20° of dorsiflexion. The distance between the location of Myoton measurement and the calcaneus had been recorded: this same distance was then located on the ultrasound scan using the calcaneus as a land-mark. The transverse scan corresponding to the recorded location of measurement was de-identified and exported to Image J (National Institute of Health, Bethesda, Maryland, USA). Antero-posterior thickness was measured following calibration of the Image J software using a Near Field Ultrasound Phantom (Model 050, Computerized Imaging Reference Systems, INC, Virginia, USA), as previously described [20]. To limit the possibility of assessor bias during ultrasound scanning (e.g. increased contact pressure resulting in decreased tendon thickness), we employed a “hands-off” approach as described above, where the ultrasound tracker was clamped into position and not manipulated by the researcher. To control for bias during analysis, images were de-identified and analyzed in a batch containing scans from tendinopathy and control groups, to ensure the researcher was blind to participant group.

### Study size

The magnitude of difference in *k*_*TR*_ between those with and without Achilles tendinopathy was, at the start of this study, unknown. Because *k*_*TR*_ and *k*_*TE*_ are, at least in some scenarios, correlated, [21] we used *k*_*TE*_ values from a study by Arya & Kulig [4] (tendinopathy subjects: 300.4 + 37.6 N/mm; control subjects: 375.3 + 61.9 N/mm) to estimate that a sample size of n = 6 tendinopathy and n = 6 controls would allow for an independent t-test with 80% power and 0.05 alpha.

### Statistical analysis

Values are presented as mean (standard deviation/SD or range). We used the SPSS statistical package (SPSS Software, Version 25.0, IMB Corporation, Armonk, New York). For between-week reliability, we calculated the intra-class correlation coefficient (ICC, two-way mixed effects, relative agreement, single rater/measurement) and minimum detectable change (individual) with 95% confidence using the formula SEM * 1.96 * √2, where SEM (standard error of measurement) represents the SD of measurement_week1_/ √(1—ICC), where n is the number of individuals in each group (15 or 10 for tendinopathy and controls respectively). We used independent t-tests with Bonferroni correction to compare the means of tendinopathy and control groups for the following variables: *k*_*TR*,_ N/m, age (yrs), years running experience, body mass index (kg/m^2^), waist circumference (cm), weekly running distance (km), average number of running days per week, VISA-A (0–100), tendon thickness (mm), tendon cross-sectional area (mm^2^). We conducted univariate Pearson correlations between *k*_*TR*_ and the following variables: age, VISA-A, tendon thickness, and waist circumference. Correlations were conducted on the combined study population, except in the case of VISA-A: completely healthy participants (VISA-A of 100) were excluded from this analysis to avoid pooling of heterogenously distributed samples [22]. We limited the number of statistical tests to those described above in order to minimize the possibility of a Type II error.

## Results

Participants were recruited from March 2018 to July 2018. A total of fifteen participants with Achilles tendinopathy were contacted for eligibility purposes and twelve participants passed the initial phone screening. Two tendinopathic participants were screened in person but not included: one participant did not engage in regular running, and the other had insertional Achilles pain. Twenty potential control participants expressed interest in the study. Of these, five voluntarily withdrew before the study began due to a busy schedule, two were not included because they did not engage in regular running, and one was not included due to age. All participants (tendinopathy n = 10, controls n = 15) who were included in the study attended both required appointments.

### Participant characteristics

The participant characteristics are shown in Table 1. All participants were recreational runners with an average weekly running distance of 38 km (SD25, range 15–125), and reported running as their main physical activity. The average age of all participants was 49 (26-71yrs) and the average BMI was 25kg/m^2^ (19–31). All tendinopathic participants reported a gradual onset of symptoms, received a physician’s diagnosis, displayed typical ultrasound appearance, presented with pain / discomfort upon palpation, and had readily observable, localized Achilles swelling. The average tendon thickness of patients was 6.8 mm (0.9), vs 5.5 mm (0.9) in controls (p< 0.001). The average VISA-A score was 69 (8.1) in the tendinopathic group and 99 (0.5) in the control group. No participant underwent physiotherapy treatment during the one week of testing, and all reported no change to their activity level or footwear within 48 hours of testing on both appointments. Sex assigned at birth, body mass, height, BMI, waist circumference, weekly running distance and number of running days per week were equivalent between groups (Table 1).

**Table 1:** Participant characteristics.

Parameter	Achilles Tendinopathy Group (n = 10)	Control Group (n = 15)	*P* Value
Gender, m/f	5/5	7/8	-
Age, years: mean (SD)	48 (8.9)	50 (15)	.82
Body Mass Index, kg/m^2^: mean (SD)	24 (3.4)	25 (2.8)	.69
Waist Circumference, cm: mean (SD)	79 (12)	83 (8.4)	.36
Weekly Running Distance, km: mean (range)	43 (15–80)	34 (8–125)	.13
Average Number of Running Days Per Week, mean (SD)	3.7 (1.5)	3.4 (1.2)	.60

P values reflect independent t-tests except for weekly running distance (Mann-Whitney U).

### Relationship between tendon stiffness and clinical parameters

**Fig 2:**
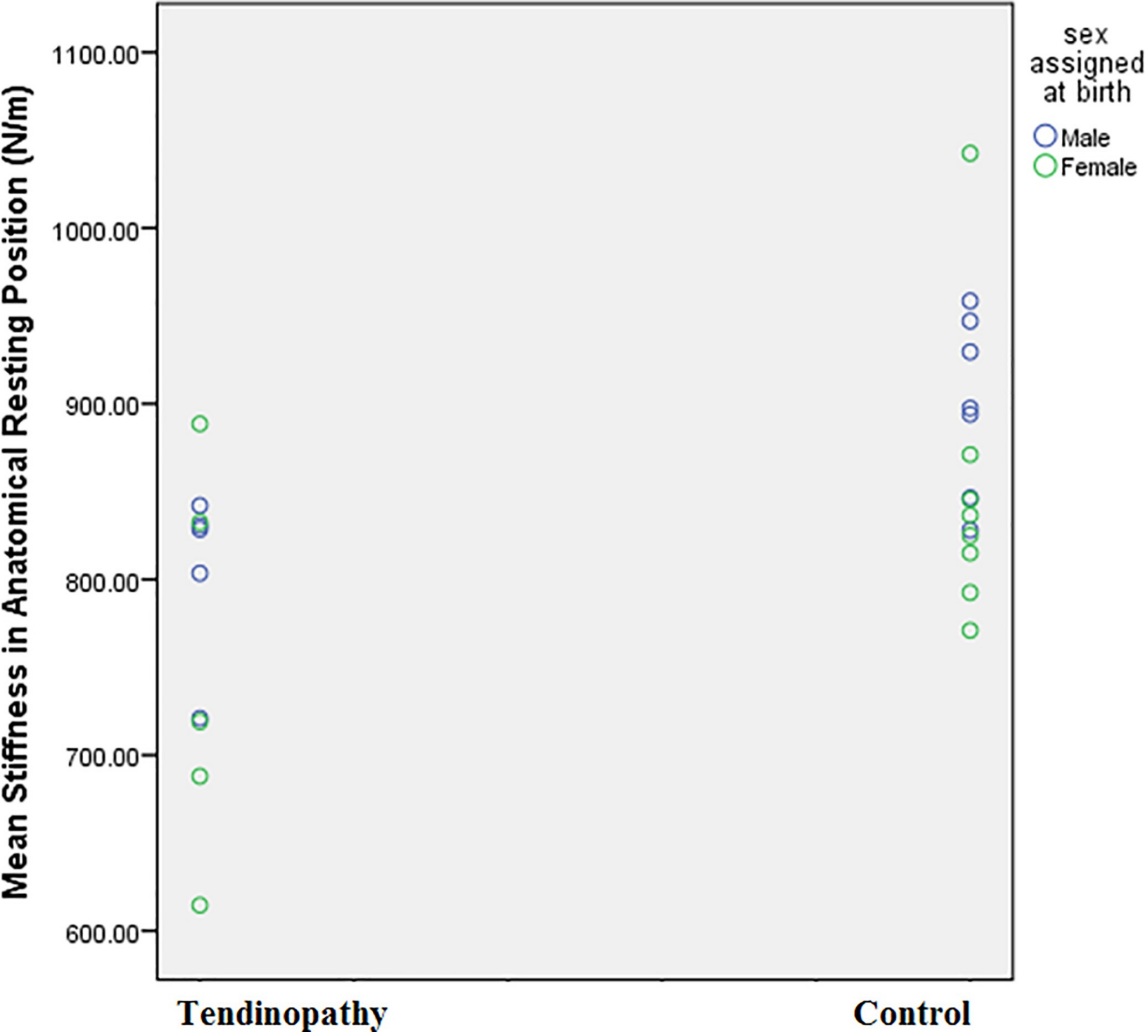
Achilles tendon transverse stiffness (*k*_*TR*_) in recreational runners with or without tendinopathy. Average stiffness was lower among patients with Achilles tendinopathy (777 N/m (86)) than controls (873 N/m (72), p < 0.05, n = 25). Black circles denote males, blue denote females.

**Fig 3:**
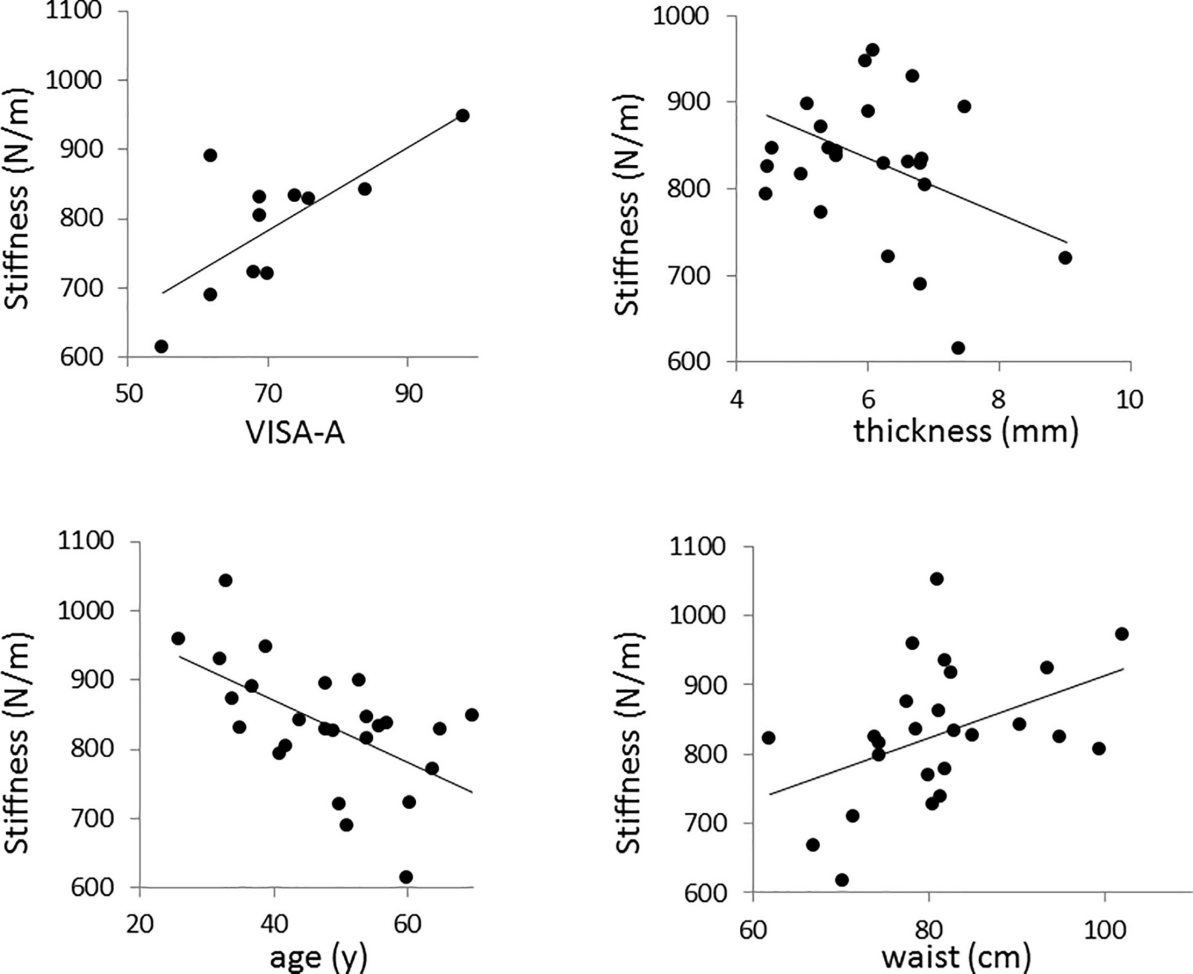
Relationships between Achilles tendon transverse stiffness (*k*_*TR*_) and clinical parameters. (A) Stiffness was positively correlated with VISA-A (r = 0.73, p = 0.011, n = 11 participants with VISA-A < 100) and (B) negatively correlated with thickness (r = -0.39, p = 0.051, n = 25). (C) Stiffness was negatively correlated with age (r = 0.51, p = 0.009, n = 25) and (D) positively correlated with waist circumference (r = 0.43, p = 0.03, n = 25).

Stiffness was lower on average in the tendinopathy group than in the control group, both in men and women (Fig 2). However, there was considerable overlap in the values with no clear cut-off which could be taken to indicate the presence of tendinopathy. Stiffness was positively correlated with VISA-A (Fig 3A) and negatively correlated with tendon thickness (Fig 3B), indicating that worse symptoms and more thickened tendons were accompanied, on average, by lower stiffness values. Older participants tended to have lower tendon stiffness values (Fig 3C). Unexpectedly, those with larger waist circumference demonstrated a tendency toward greater tendon stiffness (Fig 3D).

### Reliability of tendon stiffness in people with Achilles tendinopathy

The stiffness values demonstrated good reliability over the course of one week (Table 2).

**Table 2:** Minimum detectable change with 95% confidence (MDC95), SEM (standard error of measurement) and intraclass correlation coefficient (ICC) of tendon stiffness.

	MDC95 (N/m)	ICC	SEM
Injured (n = 10)	118	0.81	43
Healthy (n = 15)	87	0.80	32

## Discussion

Achilles tendinopathy is typically accompanied by material changes in the tendon properties including collagen fiber disorganization, increased glycosaminoglycan content, and vascular proliferation [8]. These alterations are believed to result in a functional impairment in the tendon which can then predispose to ongoing injury and nociception. In this study, we set out to describe a novel method for assessing the mechanical properties of tendinpoathic tendon tissue. The main findings of this study are (a) that transverse tendon stiffness is reduced on average in people with Achilles tendinopathy when compared to asymptomatic controls who are similar in age, gender balance, and tendon-loading habits, and (b) the stiffness values are highly reproducible, with an ICC of 0.81 for people with tendinopathy.

Prognosis of Achilles tendinopathy in the general population is not well established–many cases are probably mild, self-limiting and/or self-managed. In advanced or chronic cases where professional assistance is sought, most (94%) recover in the longterm [23], although return to full activity can be a lengthy process, with some people opting for surgery [24]. Heavy, isometric exercise is capable of inducing adaptive, material changes in tendon tissue [25]. Therefore having a measurement technique to set individualized targets–similar to testing muscle strength, but focused on tendon–could be beneficial. Currently, the responsiveness of tendinopathic Achilles tissue to exercise has not been directly assessed, and the measurement technique described here opens up the possibility of conducting that assesment. Tendon transverse stiffness could in future become a useful adjunct measure of impairment, and a way of monitoring whether tendon function is improving, worsening, or staying the same.

In the current study, we measured tendon stiffness at a single location (the most symptomatic region). Stiffness varies along the length of the tendon, becoming progressively greater from the muscle to the bone [1]; this anatomic variation may minimize an abrupt transition between the very compliant muscle and incompliant bone. In future, it might be useful to take several measures of tendon stiffness from proximal to distal, in order to gain a more complete functional profile of a person’s tendon–it could be hypothesized that stiffness will be more variable in injured tendons, and that this variability will reduce as the tendon becomes less symptomatic. At the present time, we are conducting a follow-up, longitudinal study to find out whether transverse tendon stiffness improves over time with rehabilitation.

In this study, we did not standardize the position of the foot, but simply allowed the ankle to adopt a natural, resting position. In pilot work, we systematically varied the angle of the ankle from 0° to 20° of dorsiflexion and found that (a) the rate of stiffness increase with progressive dorsiflexion was not uniform, (b) the ability to detect deficits in tendinopathic tissue disappeared, and (c) measurements became less reliable with increasing joint angle. If a clinical testing protocol is to be developed, we would echo the Myoton manufacturer’s instructions and recommend testing with the muscle and tendon on slack.

The study population included 10 people with Achilles tendinopathy and 15 without, making it rather small. Our power calculation for the comparison of tendinopathy and controls yielded a required sample size of only 6 per group with power of 80%, but because it was feasible to recruit more participants, we did so with the rationale that the larger the sample, the greater the statistical power. A future larger study including more diverse groups of Achilles tendinopathy patients would further improve the generalizability of the study.

In addition to injury, which leads to a pathological loss of tendon stiffness,[26] exercise can lead to an adaptive increase in tendon stiffness [2]. The mechanisms by which tendon stiffness increases in response to execise are not fully understood, but may involve a combination of new collagen fibre formation [27] and increased density of cross-links [28]. Longterm exercise habits are also capable of inducing changes in tendon cross-sectional area [29]. In this study, because were were interested in studying the hypothesized loss of stiffness due to injury, we attempted to control for the influence of adaptive changes by studying individuals for whom running was the main form of physical activity, and then balancing the self-reported volume of running between groups. Despite the wide variation in running behaviour (Table 1), we were able to balance the groups to some extent on this factor, which perhaps allowed us to minimize the differences in exercise-induced adaptive changes in tendon stiffness between groups.

We detected a positive correlation between waist circumference and tendon stiffness, which leads us to reject our hypothesis that there would be a negative correlation between these parameters. The rationale for hypothesizing that there would be a negative correlation was that increasing adiposity has been associated with increased risk of tendinopathy. Our study population, however, contained no obese individuals (the highest BMI was 29 kg/m^2^). Waist circumference was in fact correlated (r^2^ = 0.48) with standing height, indicating that in our population waist circumference was predominantly related to body size rather than adiposity. In this regard, it makes sense that larger individuals would have stiffer tendons, due to the training simulus of increased ground reaction forces.

This study presents a number of additional limitations. The study was cross-sectional, and relatively small: therefore, it would be useful to conduct this study in a larger group over a longer period of time, e.g. during the period before onset of symptoms, or during post-injury rehabilitation. We cannot generalize the findings to individuals with other conditions, or who are engaged in other types of activities than endurance running. We only measured a single location, didn’t compare bilateral changes, and only conducted univariate analyses. We did not obtain direct measures of adiposity. We did not attempt to derive measures of tendon internal structure (e.g. Type I echoes) from the ultrasound images, as previously we found a lack of association between ultrasound echotexture and biomechanical parameters [25].

Another potential limitation is that the measurement is influenced by the skin and subcutaneous tissue overlying the tendon.[1] Even though the influence of this tissue appears small in a controlled laboratory study,[1] in a general population there may be a wide variety of skin and body types which could interfere with the measurement of transverse tendon stiffness. For this reason, our current working model is to explore this tool as a technique to detect intra-individual changes (i.e. over time) when the tendon is expected to alter its properties, but the overlying skin is not (e.g. tendon-targeted rehabilitation).

The number of days run per week and average weekly running distance were extremely varied, in both the tendinopathy and control groups (Table 1). Nevertheless, the attempt to balance the tendinopathy and control groups according to number of days run per week and average weekly running distance may have introduced some bias into the measurements. It is possible that people with Achilles tendinopathy have different running habits than those without tendinopathy, therefore balancing on this factor could make the Myoton measures biased.

In conclusion, Achilles tendon transverse stiffness can be reliably measured using a hand-held dynamometer. Participants with Achilles tendinopathy demonstrated a lower transverse tendon stiffness than those without tendinopathy, and there were negative correlations between stiffness and age as well as stiffness and thickening, and a positive correlation with tendon function (VISA-A score). A future longitudinal study may be able to determine whether tendon stiffness improves during rehabilitation, and whether that improvement correlates with a reduction in symptom severity.

## Supporting information

S1 Dataset Complete data set for statistical hypothesis testing. (XLSX)
